# Dppa3 facilitates self-renewal of embryonic stem cells by stabilization of pluripotent factors

**DOI:** 10.1186/s13287-022-02846-8

**Published:** 2022-04-27

**Authors:** Shuang Zhao, Chuanyu Zhang, Jia Xu, Siying Liu, Lu Yu, Shang Chen, Hang Wen, Zongjin Li, Na Liu

**Affiliations:** 1 School of Medicine, Nankai University, 94# Weijin Road, Tianjin, 300071 China; 2 Key Laboratory of Bioactive Materials, Ministry of Education, College of Life Sciences, Nankai University, Tianjin, 300071 China

**Keywords:** Dppa3, Nanog, Degradation, Embryonic stem cells

## Abstract

**Background:**

Developmental pluripotency-associated 3 (Dppa3, also called Stella or PGC7) is a principal maternal protein specially expressed in pre-implantation embryos, embryonic stem cells (ES cells) and primordial germ cells (PGCs). It plays critical role in the regulating of DNA methylation in zygotes and oocytes. However, the effect of Dppa3 in ES cells on the stability of proteins is still unclear.

**Methods:**

In this study, we first identified the potential interacting proteins with Dppa3 using immunoprecipitation-mass spectrometry (IP-MS). After GO analysis, we further constructed Dppa3-silenced ES cells and ES cell lines overexpressing with different lengths of Dppa3 to explore the mechanisms of Dppa3 on protein stability.

**Results:**

IP-MS results showed that Dppa3 interacted with quite a few subunits of 26S proteasome. Full length of Dppa3 stabilized Uhrf1 and Nanog by inhibiting its degradation. Silencing Dppa3 promoted degradation of Nanog protein.

**Conclusions:**

Our results indicated that Dppa3 safeguard the stability of Uhrf1 and Nanog by inhibiting proteasome-associated degradation in ES cells. These findings shed light on new function of Dppa3 in maintaining stability of proteins and provides a valuable resource for understanding the roles of Dppa3 in embryonic stem cells.

**Supplementary Information:**

The online version contains supplementary material available at 10.1186/s13287-022-02846-8.

## Introduction

Embryonic stem cells (ES cells) are derived from the inner cell mass (ICM) of the blastocyst and can be maintained pluripotency indefinitely [8]. With the ability of self-renewal and potential differentiation to multiple cell lineages, human ES cells hold great promise for the treatment of patients with degenerative disorders [33]. Developmental pluripotency-associated protein 3 (*Dppa3*, also known as Stella or PGC7) is initially identified as a maternal gene preferentially expressed in germ cells and early embryos [29]. Dppa3 plays crucial roles in early embryonic development by modulating transcriptional program and regulating epigenetic modification [20, 23]. *Dppa3*^−/−^ male mice showed normal fertility when mated with wild-type (*Dppa3*^+/+^) or heterogenetic (*Dppa3*^±^) females. However, the pre-implantation development of embryos derived from *Dppa3*^−/−^ oocytes is abnormal, suggesting that maternally supplied Dppa3 is important in the cleavage stages of pre-implantation development [26]. However, the epigenetic behavior of Dppa3 is quite different on paternal genome and maternal genome in the zygotes. After fertilization, paternal genome is demethylated by Tet3 (ten-eleven translocation 3). Dppa3 specifically binds to the maternal genome on histone H3K9 dimethylation (H3K9me2) and protects the maternal genome against Tet3-mediated conversion of 5mC to 5hmC during the wave of DNA demethylation in early embryogenesis [22, 23, 26, 29] and the mechanism of Dppa3 on DNA methylation in zygotes is distinguish different with that in oocytes, suggesting the complexity functions of Dppa3 in different development stages [14]. Shin et al*.* found that the cleavage of full length of Dppa3 is also necessary for early embryonic development [32].

In addition to the function of Dppa3 in embryogenesis, it is also heterogeneously expressed in embryonic stem cells. Dppa3 is required for generation of fully reprogrammed induced pluripotent stem cells (iPS cells), and increases the efficiency of nuclear transfer reprogramming [5, 12, 14, 22, 36, 41]. With the development of single-cell analysis technology, ES cells display heterogeneous and metastable state that fluctuate between naïve state and primed state [21, 24]. A defined medium known as “2i medium” (MEK inhibitor: PD0325901, and GSK3 inhibitor: CHIR99021) contributes to the establishment of a ground pluripotent state in ES cells, which renders them similar to the cells of the inner cell mass [25, 43]. In contrast, primed ES cells are similar to epiblast stem cells (EpiSCs), which can be induced by FGF2 and Activin A (FGF + ActA) [6]. Compared with primed ES cells, ground or naïve ES cells have a higher level of Dppa3 expression [12, 28]. Epigenome in Dppa3-positive ES cells are similar to that in ICM, characterized with hypomethylated DNA [12, 28, 46]. The function pattern of Dppa3 in ES cells is different from embryo. In ES cells and somatic cells, Dppa3 causes the demethylation of whole genome [7, 9]. Dppa3 is also critical for the maintenance of Dlk-Dio3 imprinting [13, 41].

The stability of proteins in all activities remains controllable balance. There are two main degradation systems in cellular activities. One is autophagy–lysosome system. The other is ubiquitin–proteasome system (UPS) [27]. UPS plays a significant role in zygotic gene activation, DNA repair, and developmental programs [4, 10, 30, 31] Dppa3 is involved in degradation of proteins in early embryogenesis, but the role of which in regulating protein stability in ES cells remains poorly understood. Here, we demonstrated that Dppa3 regulates proteins stability in ES cells. Specifically, we found that the accumulation of Uhrf1 in ES cells with full length of Dppa3 overexpression. Furthermore, Dppa3 overexpression also increased Nanog in ES cells. This provides new insights into the function of Dppa3 in ES cells.

## Materials and methods

### Cell culture

Mouse ES cells were all cultured in high glucose DMEM (Gibco, Thermo Fisher Scientific ™) supplemented with 15% fetal bovine serum (Hyclone), 2 mM L-glutamine (Gibco, Brazil), 5000U/mL penicillin and streptomycin (Gibco, USA), 0.1 mM NEAA (Gibco, USA), 0.1 mM 2-Mercaptoethanol (Sigma), and 1000U/ml LIF (ESG1107, Millipore Crop, USA) at 37 °C in 5% CO_2_ incubator. Fibroblast cells are cultured in DMEM supplemented with 10% fetal bovine serum (Biological Industries), 2 mM l-glutamine (Gibco, Brazil), 5000 U/mL penicillin and streptomycin (Gibco, USA). 2i/LIF conditional culture medium contains DMEM/F12 (Gibco, Thermo Fisher Scientific ™), Neurobasal (Gibco, USA), N2 (Gibco, USA), and B27 (Gibco, USA) medium supplemented with PD0325901 (Sigma) and CHIR99021 (Sigma). FGF/Activin A conditional culture medium contains DMEM/F12 (Gibco, Thermo Fisher Scientific ™), Neurobasal (Gibco, USA), N2 (Gibco, USA), B27 (Gibco, USA) medium supplemented with FGF2 (Pepro Tech), and Activin A (Pepro Tech).

### Plasmid construction and establishment of Dppa3 overexpression cell lines

We used pCMV6-entry (OriGene Technologies) to amplify *Flag* sequence first and then cloned it into plasmid plch3.7. Primers were linked with restriction enzymes Nhe I (Takara) and Psp5 II (Takara) for further enzymatic digestion. Then, introduce plch3.7-Flag as scaffold for plasmids construction. Mouse *Dppa3* CDS contains 450 base pairs. 1–447, 1–180, and 181–447 base pairs (except for the last termination codon) were cloned into plch3.7-Flag with restriction enzymes Nhe I (Takara) and Xho I (Takara). Primers of three different lengths of *Dppa3* are shown in Additional file 1: Table S1. All three different lengths of *Dppa3* were followed by *Flag* sequences. We named 1–447, 1–180, and 181–447 of *Dppa3* plasmids as plDf, plD1, and plD61. Plasmid plch3.7 is a load control compared to other three plasmids. D3 wild-type embryonic stem cell lines and 293 T cell lines were transfected with plasmids mentioned above using Lipo 2000 reagent (Invitrogen) with serum-free medium Opti-MEM I® according to the manufacturer’s instructions. The cells were cultured by non-penicillin or streptomycin culture medium for 2 days. Transfected ES cells and 293 T cells were then selected by puromycin (0.5 μg/ml) for 10–14 days. For transfected ES cells, individual clones were picked for prolong culture and analysis. For transfected 293 T cells lines, the protein level of Flag was evaluated by western blot assay.

### Generation of Dppa3 KD ES cells

shRNAs (Additional file 1: Table S2) were synthesized and cloned into pSIREN-RetroQ vector. The reconstructed vectors were transfected into ESCs using Lipofectamine 2000 (Invitrogen). ESCs were selected by puromycin (1.5 μg/ml) for 7 days, and positive clones were next used for analysis.

### Immunoblots

Cell were lysed with RIPA (Cat: R0020, Solarbio) supplemented with protease inhibitor (Sigma). Proteins were separated on 8–12% SDS gels and electrophoretically transferred to PVDF membranes (Cat: IPVH00010, Millipore, 0.22 μm). Using 5% Difco ™ skim milk (BD, France) to block signals nonspecifically for around 2 h at room temperature before primary antibodies were incubated overnight at 4℃. Secondary antibody was incubated at room temperature for 1–2 h after washing primary antibodies. The following antibodies were used: Dppa3 (1:1000, Cat: ab19878, Abcam); Tubulin (1:1000, Cat:M20005S, Abmart); H3 (1:1000, Cat: ab1791, Abcam); DYKDDDDK-Tag (3B9) (1:5000, M20008, Abmart); and Nanog (1:1000; Bethyl).

### Real-time polymerase chain reaction

Total RNA was isolated by TRIzol™ Reagent (Thermo Fisher Scientific™) and reversely transcribed into cDNA from 1 μg of total RNA using Transcriptor First Strand cDNA Synthesis Kit (Roche). Real-time polymerase chain reaction (RT-PCR) was performed with the Opticon® System (Bio-Rad, Hercules, CA) using Hieff™ qPCR SYBR® Green Master Mix (No Rox) (Yeasen) to amplify expressions of relative genes. Relative gene expression fold change was identified using the 2^−ΔΔCt^ method. The sequences of primers used in this study are shown in Additional file 1: Table S3.

### Alkaline phosphatase staining

ES cells were seeded at a density of 5 × 10^4^ cells per well into 24-well plates and cultured for one passage. Alkaline phosphatase (AP) staining was performed with the Alkaline Phosphatase Staining Kit (Yeasen). The cells were fixed with 4% paraformaldehyde for 10 min after washing with PBS and incubated with AP solution at room temperature for 10–15 min. PBS was then added to terminate the reaction. The stained cells were observed and recorded for the number of colonies under the microscope. Data were analyzed by counting at least 300 colonies for each sample.

### CCK-8 proliferation assay

To investigate whether overexpressing different lengths of Dppa3 could affect the proliferation of ES cells, the ES cells were seeded into 96-well plates at a density of 10^4^ cells per well for each overexpression cell lines. After 12 h, the reagent of Cell Counting Kit-8 (Solarbio) was added to the medium and incubated for an additional 1 h. The absorbance value of each well was recorded at 450 nm using a microplate reader (Thermo Labsystems, Vantaa, Finland).

### Immunofluorescence staining

Cells were fixed in 4% paraformaldehyde for 20 min and incubated at 4 °C in blocking buffer (PBS containing 5% BSA). Cells were incubated in the presence of primary antibodies including Dppa3 (1:1000; Abcam) and Nanog (1:1000; Bethyl) at 4 °C overnight and then washed three times in PBS. Cells were then incubated with Alexa Fluor 488 secondary antibody (1:1000; Invitrogen) for 2 h at room temperature. Nuclei were stained with DAPI (1:10,000; Invitrogen).

### Immunoprecipitation

ES cells were scrapped off the 10 cm flasks and centrifuged (800 × *g*, 5 min). After being washed twice with cold 1 × PBS, cells were lysed for 15 min on ice in IP buffer (50 mM Tris, pH 7.4, 150 mM NaCl, 10% glycerol, 1% Triton X100, 0.5 mM EDTA, 10 mM NaF, 100 μM orthovanadate, 200 μM PMSF). The cell lysates were clarified by centrifugation (15,000 × *g*, 4 °C, 15 min), and the supernatant was incubated with anti-FLAG M2 affinity Gel (Sigma) at 4 °C with rotation. The complexes were precipitated, washed twice with IP lysis buffer supplemented with 500 mM NaCl, and then washed six times with IP lysis buffer alone. Bound complexes were eluted with FLAG peptide (100 μg/ml) (Sigma) and separated by SDS-PAGE (10%) after boiling (5 min, 95 °C) in 1 × SDS sample buffer. Coomassie blue stain of whole band was isolated and identified by LC–MS/MS.

### Flow cytometry analysis

Colonies were dissociated into single cells and resuspended in corresponding culture medium at a density of approximately 5 × 10^6^ cells/ml. Cells were fixed and permeabilized by 3.7% paraformaldehyde and 0.1% Triton X-100 in PBS, blocked by 4% BSA, and then incubated with primary antibodies including Dppa3 (1:500; Abcam), Nanog (1:500; Bethyl), or isotype-matched negative control. Alexa Fluor 594 goat anti-mouse IgG (6 μg/ml; Invitrogen) was used as secondary antibodies. Cells were then analyzed using FACSAria Cell Sorter (FACStar Plus Flow Cytometer; Becton-Dickson).

### Hematoxylin–Eosin staining

Dppa3 overexpression ES cells and Dppa3 knockdown ES cells were injected into nude mice for teratoma analysis. After 4 weeks, the nude mice were killed and the teratoma was collected for the HE staining according to standardized procedures. Teratoma was washed in PBS, fixed in 4% paraformaldehyde (pH 7.4), and embedded in paraffin. Teratoma was stained using hematoxylin for 5–10 min, rinsed with distilled water for 1 min, and separated color with 0.5% alcohol hydrochloric acid at 37 °C. Teratoma was then stained with eosin for 2–5 min, dehydrated using graded ethanol, vitrified by dimethylbenzene, and mounted with neutral balsam at 37 °C.

### Statistical analysis

Data were analyzed by Student’s t test using SPSS software. Statically significant differences were defined as *p < 0.05, **p < 0.01, ***p < 0.001. The results were shown as mean ± SEM.

## Results

### Identify Dppa3-interacted proteins in embryonic stem cells

**Fig. 1:**
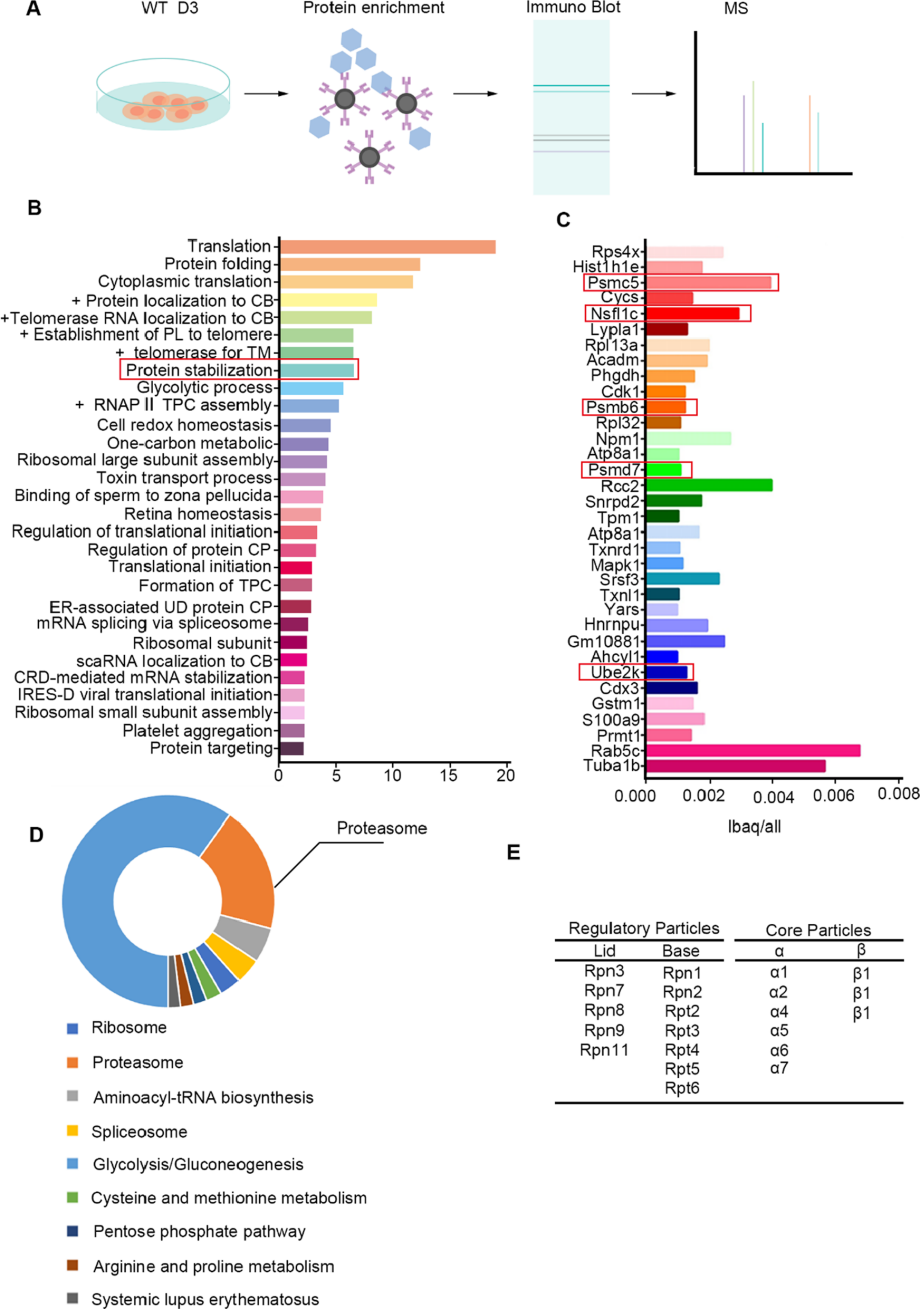
Identify Dppa3-related proteins using IP-MS. **A** Schematic representation of immunoprecipitation-mass spectrum (IP-MS) technique. Enrichment of Dppa3-interacted proteins using immunoprecipitation with anti-Dppa3 antibody. And then the Dppa3-interacted proteins were analyzed by MS. **B** The pull-down proteins were mainly participant in translation, protein folding and cytoplasmic translation, etc. +, Positive regulation. CB, Cajal body. PL, Protein localization. TM, Telomere maintenance. TPC, Transcriptional preinitiation complex. CP, Catabolic process. UD, Ubiquitin-dependent. CRD, Coding region instability determinant. IRES-D, Internal ribosome entry site-dependent. **C** The list shows some of Dppa3-interacting proteins ranked by ibaq/all. **D** KEGG analysis of IP-MS results using DAVID database. **E** A list of components 26S proteasome which have interactions with Dppa3

Dppa3 is heterogenetic expressed in ES cells and plays important role in somatic cell reprogramming. To further understand the mechanisms of Dppa3 in ES cells, we firstly identified the proteins which interact with Dppa3 using immunoprecipitation-mass spectrum (IP-MS) after the cell lysis was enriched by Dppa3 antibody (Fig. 1A). GO results show that the Dppa3-interacted proteins were mainly involved in post-translational modification, ribosomal structure and biogenesis, protein transportation, coenzyme transport and metabolism, and energy production (Additional file 1: Fig. S1A). Using STRING website, we found that several Dppa3-interacted proteins play critical important roles in the pluripotency of ES cells, such as Dppa2/4, Uhrf1, Pou5f1, and Nanog [15, 19] (Additional file 1: Fig. S1C). We analyzed the function enrichment of Dppa3-interated proteins using DAVID website. The results showed that Dppa3 is involved in the regulation of protein stabilization (Fig. 1B). KEGG analysis turned out that Dppa3 interacts with many of proteasome-associated proteins (Fig. 1D) as well as proteins function in post-translational modification (Additional file 1: Fig. S1B). For instance, Psmc5, Nsflc, Psmb6, Psmd7, and Ube2k [16, 44] are components of ubiquitin proteasome system, which participate in UPS-mediated degradation of protein (Fig. 1C). Our results showed that Dppa3 also interacts with quite a few subunits of 26S proteasome (Fig. 1E), suggesting Dppa3’s potential role in regulating protein stability in ES cells.

### Full length of Dppa3 stabilizes Uhrf1 in ES cells

**Fig. 2:**
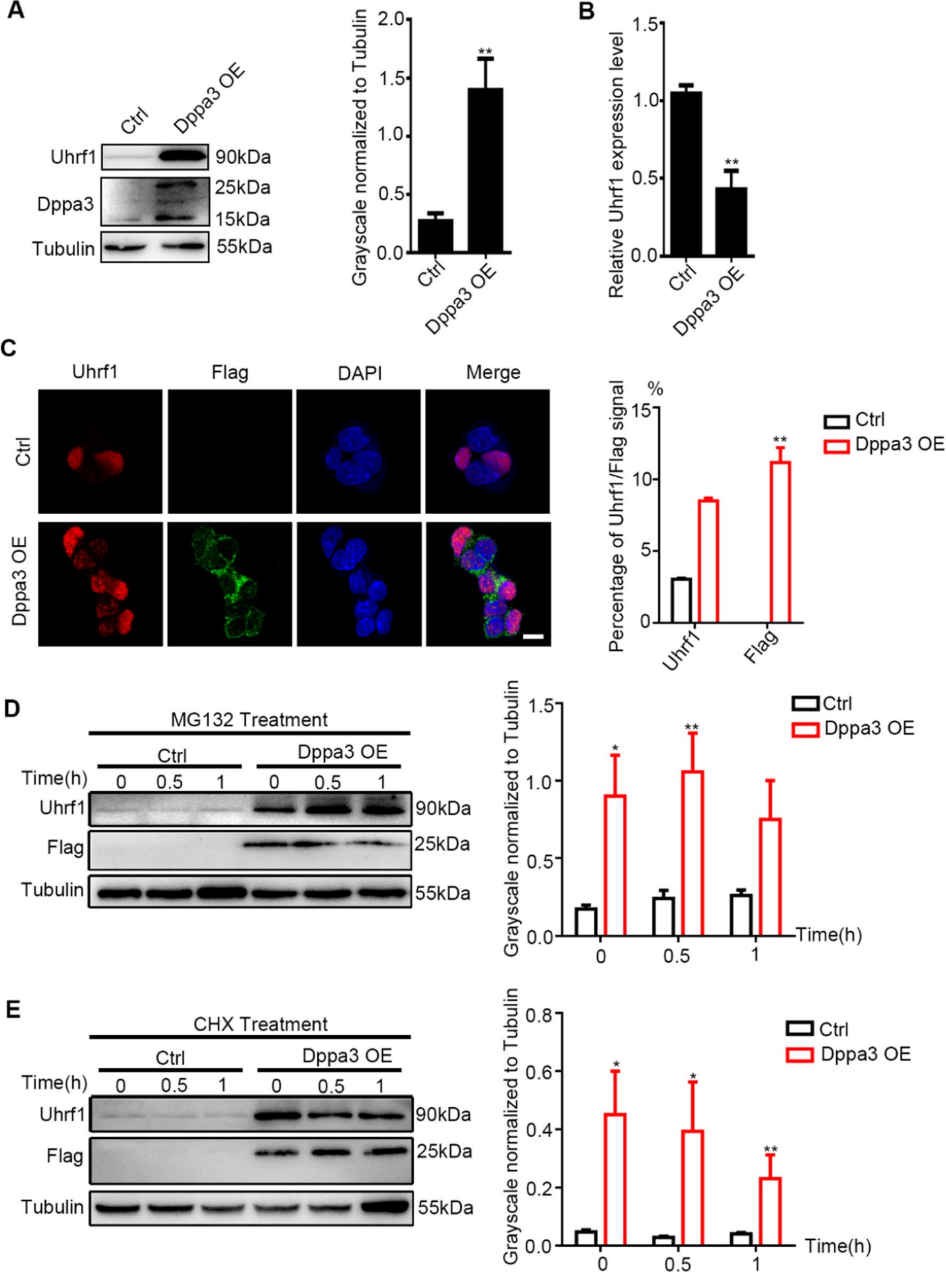
Dppa3 inhibits the degradation of Uhrf1 in embryonic stem cells. **A** Left panel: western blot analysis of Uhrf1 and Dppa3 protein levels in control group and Dppa3 overexpression group. Molecular masses (kDa) on right. Right panel: Quantification of Uhrf1 protein level normalized to Tubulin by the ImageJ software. The data are presented as mean ± SEM. (*n* = 3; **p < 0.01). **B** Quantitative RT-PCR analysis of *Uhrf1* expression in control and Dppa3 overexpression embryonic stem cells group. The data are presented as mean ± SEM. (*n* ≥ 3; **p < 0.01). **C** Left panel: immunofluorescence staining of Flag and Uhrf1 in control and Dppa3 overexpression embryonic stem cells group under the serum plus LIF condition. Red: Uhrf1. Green: Flag. Scar bar: 200 μm. Right panel: percentage of Uhrf1 and Flag signal. **D** Left: Western blot analysis of Uhrf1 and Flag protein levels in control group and Dppa3 overexpression embryonic stem cells group at the indicated time points (0 h, 0.5 h, 1 h) after MG132 treatment (20 mM). Right: Quantification of Uhrf1 protein level normalized to Tubulin by the ImageJ software. The data are presented as mean ± SEM. (*n* = 3; **p < 0.01). **E** Left: Western blot analysis of Uhrf1 and Flag protein levels in control group and Dppa3 overexpression embryonic stem cells group at the indicated time points after CHX treatment (2 mM). CHX, cycloheximide. Right: Quantification of Uhrf1 protein level normalized to Tubulin by the ImageJ software. The data are presented as mean ± SEM. (n = 3; **p < 0.01)

To investigate the role of Dppa3 on protein stability, we established Dppa3 overexpression ES cells (Dppa3 OE) and found that Uhrf1 was significantly increased in Dppa3 OE cells (Fig. 2A). However, the mRNA level of Uhrf1 was reduced in Dppa3 OE cells compared to control ES cells (Fig. 2B), suggesting that Dppa3 might regulate Uhrf1 at protein level. Immunofluorescence results also confirmed that Dppa3 overexpression increased Uhrf1 positive cells (Fig. 2C). The expression of Dppa3 was upregulated in ground-state ES cells which were induced by 2i/LIF cultural medium and Uhrf1 has similar expression pattern with Dppa3, further indicating the potential regulation function of Dppa3 to Uhrf1 (Additional file 1: Fig. S2B and C).

Next, we investigated the effects of Dppa3 on protein synthesis and degradation process of Uhrf1 using MG132 (a proteasome inhibitor) and CHX (cycloheximide, a eukaryote protein synthesis inhibitor), respectively. When the proteasome was inhibited using MG132, Uhrf1 generally accumulated from 0 to 1 h in Dppa3 overexpression group (Fig. 2D). After CHX treatment, Uhrf1 degradation was significantly inhibited in Dppa3 OE ES cells (Fig. 2E).

**Fig. 3:**
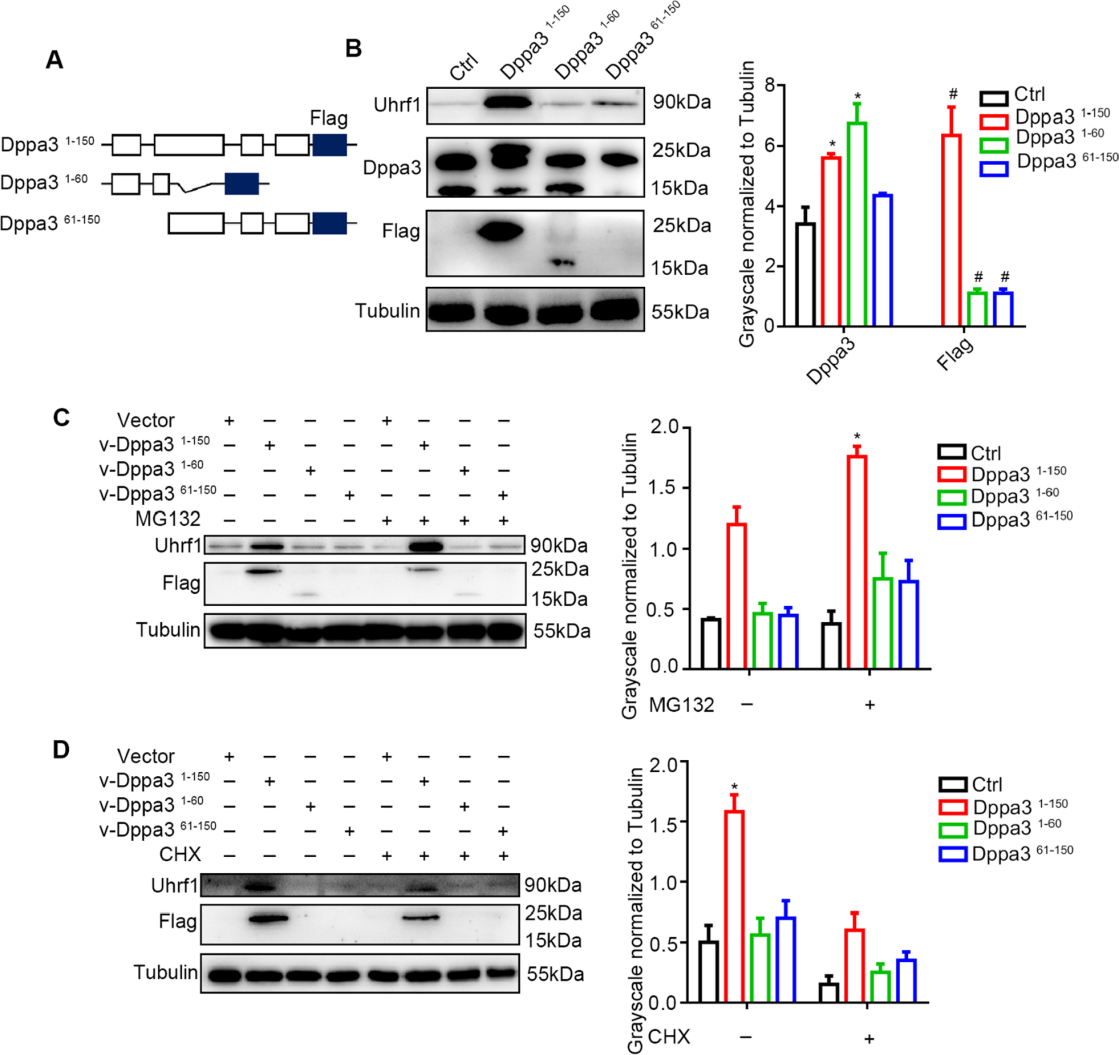
Full length of Dppa3 stabilizes Uhrf1. **A** Schematic representation of genomic overexpression of *Dppa3* tagged with *Flag*. Four exons of *Dppa3* are shown in the white box. There are three truncated versions of *Dppa3*; the first is a full length of *Dppa3* (coding 1–150 amino acids) tagged with *Flag*; the second is a truncated *Dppa3* (coding 1–60 amino acids) tagged with *Flag*; and the third is a truncated *Dppa3* (coding 61–150 amino acids) tagged with *Flag*. Three cell lines were marked as Dppa3^1–150^, Dppa3^1–60^, and Dppa3^61–150^. *Dppa3* (white box), *Flag* (blue box). **B** Left: Western blot analysis of Uhrf1, Dppa3, and Flag protein levels in ES cells with overexpression of Dppa3. Molecular masses (kDa) on right. Right: quantification of relative Dppa3 and Flag protein levels normalized to Tubulin by the ImageJ software. The data are presented as mean ± SEM. (*n* ≥ 3; *p < 0.05). **C** Left: Western blot analysis of Uhrf1 and Flag protein levels in control group, Dppa3^1−150^, Dppa3^1−60^ and Dppa3^61−150^ after MG132 treatment (20 mM). Right: Quantification of Uhrf1 protein level normalized to Tubulin by the ImageJ software. The data are presented as mean ± SEM. (*n* = 3; *p < 0.05). **D** Left: Western blot analysis of Uhrf1 and Flag protein levels in control group, Dppa3^1−150^, Dppa3^1−60^ and Dppa3^61−150^ after CHX treatment (2 mM). Right: Quantification of Uhrf1 protein level normalized to Tubulin by the ImageJ software. The data are presented as mean ± SEM. (*n* = 3; *p < 0.05)

IP-MS results showed that Dppa3 might involve in UPS-mediated protein degradation. The whole length of Dppa3 (150 amino acids) is cleaved into two truncated forms (N-terminus of Dppa3 (1–60 AA) and C-terminus of Dppa3 (61–150 AA)) in embryogenesis after fertilization. N-terminus of Dppa3 participates in vesicle trafficking along with the early embryo development, and the C-terminus of Dppa3 is easily degraded by UPS after cleavage [32], as seen in our results. Next, we constructed ES cell lines overexpression with full length of Dppa3 (Dppa3^1−150^) and two truncated versions of Dppa3 (Dppa3^1−60^ and Dppa3^61−150^), respectively (Fig. 3A). It is shown in western blot that only full length of Dppa3 increased the protein level of Uhrf1 (Fig. 3B). Next, we used MG132 and CHX to study the regulation mode of three different lengths of Dppa3 on Uhrf1. Uhrf1 protein in MG132 (20 mM, 1 h) treated group overexpression of full length Dppa3 was higher than the other two truncated groups (Fig. 3C). When treat - Dppa3^1−150^ group with CHX in concentration of 2 mM for 1 h, Uhrf1 protein still showed higher level than control group (Fig. 3D), suggesting only the full length of Dppa3 could stabilize Uhrf1 protein. This conclusion is also validated in 293 T cell lines (Additional file 1: Fig. S3A and B).

### Dppa3 stabilizes Nanog from degradation in embryonic stem cells

**Fig. 4:**
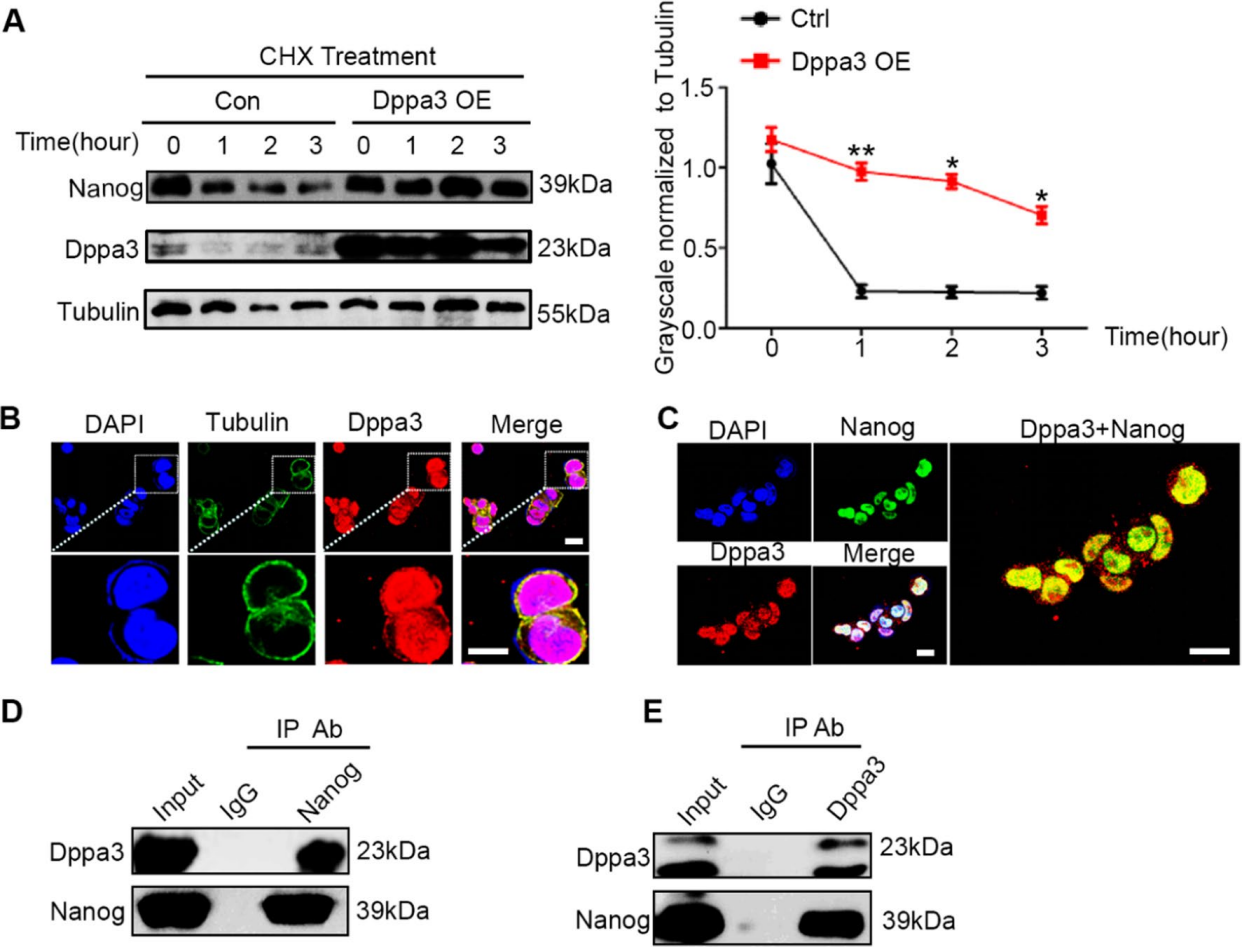
Dppa3 prevents Nanog degradation in ES cells. **A** Left panel: The protein levels of Nanog in control and Dppa3 OE ES cells were analyzed at the indicated time points (0 h, 1 h, 2 h, 3 h) after CHX treatment (2 mM). Right panel: Quantification of Nanog protein levels normalized to Tubulin by ImageJ software. The data are presented as mean ± SEM. (*n* = 3; *p < 0.05, **p < 0.01). **B** Immunofluorescence staining assay represents the location of Dppa3 in ES cells. Scale bar = 30 μm. **C** IF assay the co-localization of Dppa3 and Nanog in ES cells. Scale bar = 30 μm. **D**, **E**. Immunoprecipitation analysis of the interaction between Nanog and Dppa3 in ES cells using anti-Nanog antibody (**D**) and anti-Dppa3 antibody (**E**), respectively

Stability of proteins regulated by Dppa3 not only observed in Uhrf1, but also discovered in pluripotent transcriptional factor Nanog. Dppa3 overexpression increased Nanog expression in ES cells (Additional file 1: Fig. S4A). As shown in Fig. 4A, after CHX treatment, the protein level of Nanog in control group was extremely degraded within 1 h. However, overexpression of Dppa3 in ES cells significantly retarded the degradation of Nanog protein. This result indicated that Dppa3 stabilized the Nanog in ES cells. Immunofluorescence results indicated that Dppa3 was mainly expression in nucleus of ES cells (Fig. 4B), and co-localized with Nanog in nuclear (Fig. 4C). We also have identified the interaction between Dppa3 and Nanog by means of co-IP when used antibody against Nanog and Dppa3, respectively (Fig. 4D, E), suggesting Dppa3 might stabilize Nanog by direct or indirect interaction.

Nanog is a well-known pluripotent factor. We next investigate the pluripotency of Dppa3 OE ES cells. Our data showed that full length of Dppa3 could increase the expression of ground-state marker genes (Additional file 1: Fig. S4C), while all three different lengths of Dppa3 have no effect on proliferation (Additional file 1: Fig. S4B). Furthermore, full length of Dppa3 could increase the percentage of AP (alkaline phosphatase, a marker of pluripotency) positive clones (Additional file 1: Fig. S4D); these results indicate Dppa3, especially the full length of Dppa3, facilitates the pluripotency maintenance of ES cells.

### Silencing of Dppa3 promotes degradation of Nanog protein

**Fig. 5:**
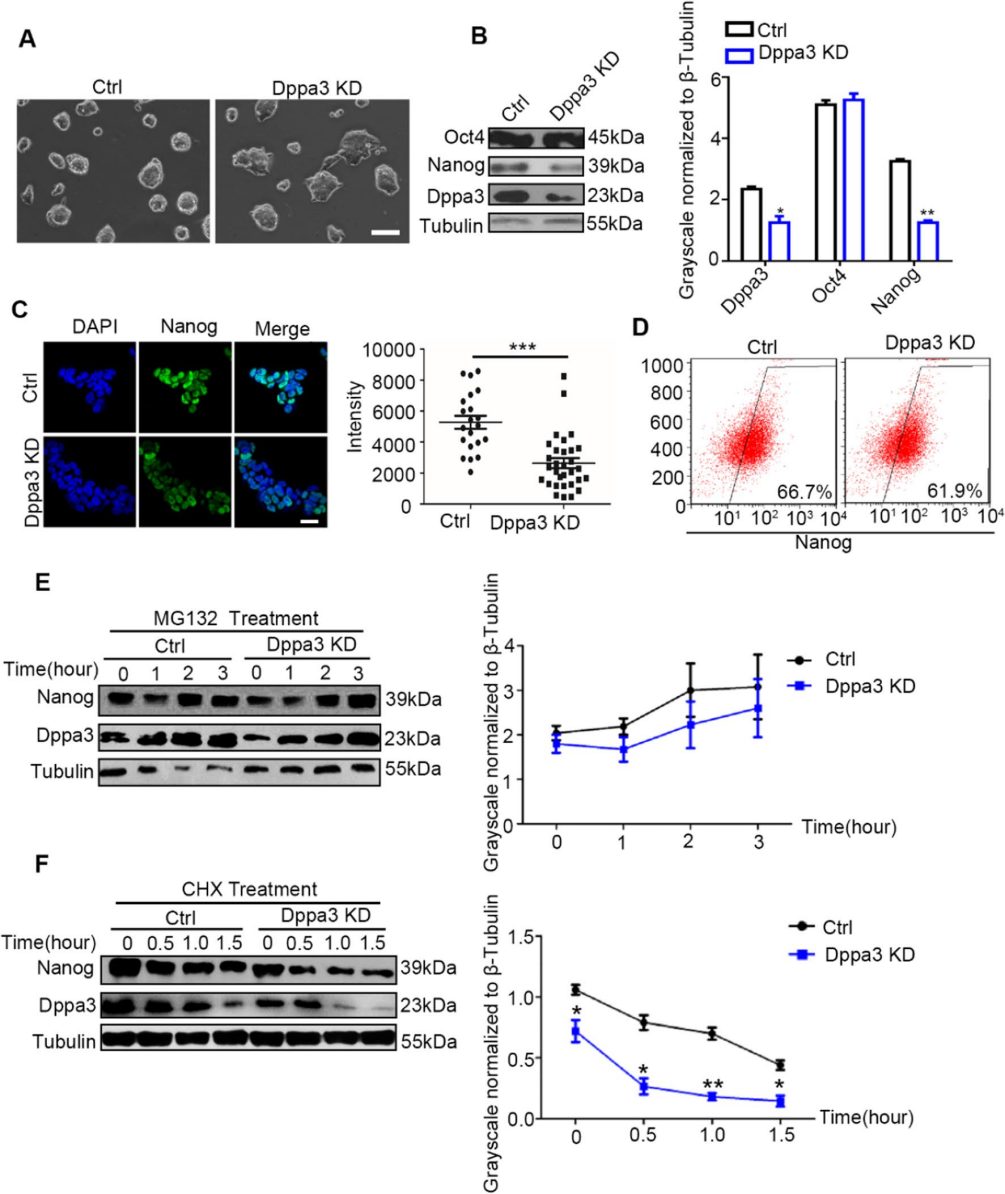
Silencing of Dppa3 downregulated Nanog expression. **A** Morphology of control ES cells and Dppa3 knockdown ES cells. Scale bar = 100 μm. **B** Left panel: Western blot analysis of Oct4, Nanog and Dppa3 protein levels in control and Dppa3 knockdown ES cells. Right panel: Quantification of Oct4, Nanog and Dppa3 protein levels normalized to Tubulin by the ImageJ software. The data are presented as mean ± SEM. (*n* = 3; *p < 0.05, **p < 0.01). **C**. Left panel: Immunofluorescence images of Nanog expression in control and Dppa3 knockdown ES cells. Scale bar = 100 μm. Right panel: Intensity analysis of every specific cell by image J. **D** Flow cytometry analysis of Nanog positive cells in control ES cells and Dppa3 knockdown ES cells. **E** Left panel: The protein levels of Nanog in control and Dppa3 knockdown ES cells were analyzed at the indicated time points (0 h, 1 h, 2 h, 3 h) after MG132 treatment (20 mM). Right panel: Quantification Nanog protein levels normalized to Tubulin by ImageJ software. The data are presented as mean ± SEM. **F** Left panel: Nanog protein levels were analyzed at the indicated time points (0 h, 1 h, 2 h, 3 h) after CHX treatment (2 mM). Right panel: Quantification of Nanog protein levels normalized to Tubulin by the ImageJ software. The data are presented as mean ± SEM. (*n* = 3; *p < 0.05, **p < 0.01)

To further verify the effect of Dppa3 on the degradation of Nanog in ES cells, we established Dppa3 knockdown (Dppa3 KD) ES cell line and verified by RT-PCR (Additional file 1: Fig. S5A). We next examined the expression level of Nanog in *Dppa3* KD ES cells. Nanog protein levels were reduced in *Dppa3* KD ES cells using western blot and IF analysis (Fig. 5B, C). By contract, Oct4 keep unchanged in Dppa3 knockdown cells (Fig. 5B). Nanog is heterogenetically expressed in ES cells, with a reduced percentage in *Dppa3* KD ES cells compared with the level in control ES cells (61.9% vs 66.7%) (Fig. 5D). Though the ability of Nanog synthesis in *Dppa3* KD cells keep unchanged (Fig. 5E), Nanog protein was extremely decreased within 1 h in *Dppa3* KD ES cells (Fig. 5F). Knockdown of *Dppa3* has no effect on the proliferation of ES cells (Additional file 1: Fig. S5B). The clone displays flatter morphology in *Dppa3* KD cell line than control group (Fig. 5A). AP staining showed decreased AP-positive clones in *Dppa3* knockdown cells (Additional file 1: Fig. S5C). Real-time PCR experiment showed silencing *Dppa3* results in a massive downregulation of ground-state markers (Additional file 1: Fig. S5D). In teratoma forming experiment, *Dppa3* knockdown cell line can only form smaller teratoma with undeveloped ectoderm, mesoderm, or endoderm (Additional file 1: Fig. S5E and F). These results indicated that silencing of *Dppa3* promotes degradation of Nanog protein and impairs pluripotency of ES cells.

## Discussion

Dppa3 is a maternal gene required for oogenesis and early embryogenesis, but which has different mechanisms on the regulation of DNA methylation in zygotes and oocytes [3, 5, 18, 22]. Besides of the embryos and oocytes, Dppa3 is also reported to regulate the methylation level in other kind of cells as well [37]. Enforced Dppa3 in NIH3T3 could trigger global DNA demethylation by interacting with Uhrf1 [9]. In some cancer cells Dppa3 could also mediate demethylation by preventing Tet3 function [37]. A recent research showed that Dppa3 promotes tumor oncogenic dedifferentiation through remodeling DNA methylation state [42].

In addition to the effects in embryos and oocytes, Dppa3 is also specifically expressed in embryonic stem cells and plays critical role in the regulation of DNA methylation and pluripotency in ES cells. Dppa3 facilitates germline and endodermal differentiation of human ES cells [40]. Our previous study also showed that Lin28a induced primed ES cells by downregulating Dppa3 [28]. In early embryos, Dppa3 is cleaved into two fragments. The cleaved maternal Dppa3 protein is essential for early embryogenesis by vesicular trafficking [32]. However, it is unclear whether Dppa3 impacts protein stability in ES cells. Here, our data showed Dppa3 interacted with regulatory particle non-ATPase (Rnp8, Rnp11, Rnp1) and regulatory particle triple-ATPase (Rpt2, Rpt6, Rpt5, Rpt3) and almost all α subunits (α1, α2, α4, α5, α6, α7) and β1, β5, and β6 subunits (Additional file 1: Fig. S1D). Ubiquitin is covalently attached to target proteins to signal their degradation by 26S proteasome [38]. UPS provides dominated regulation mechanisms within nucleus. Degradation mediated by lysosome exists in cytoplasm. It is vital for removing misfolded and damaged proteins, and proteins whose functions are no longer needed. UPS is also involved in cell cycle progression, metastasis, DNA damage, and regulatory network in diseases [34, 39]. Moreover, UPS is required for activating biological function in early embryonic development, because female pronuclear accumulates thousands of maternal proteins during the oogenesis which are essential for cleavage stage. During early embryonic development stage, numerous proteins undergo different ways to ensure the right path through development period.

Our results also indicate that Dppa3 protects Uhrf1 and Nanog from degradation in ES cells, which may provide further understanding of the role Dppa3 paly in the ES cells. Forced expression of Dppa3 in ES cells stabilized Nanog and Uhrf1, in which Dppa3 might serve as an ubiquitinated substrate to the inner catalytic chamber within the *20S* core particle and is cleaved, which may prevent other substrate to be bind by proteasome and stop the following degradation. Several evidences have shown the crystal structure of Uhrf1 binds with hemi-DNA to recruit Dnmt1 toward the replicable foci [1, 2, 11]. The Uhrf1 C-terminal RING finger functions as an ubiquitin E3 ligase to cause histone H3 ubiquitination at site of Lys18 and/or Lys23, which is subsequently recognized by Dnmt1 to promote its localization onto replication foci [17]. Degradation of Uhrf1 is mediated by ubiquitin, subsequently Uhrf1 is submitted to 26S proteasome for degradation. The existence of Dppa3 takes over the domain of Uhrf1 that is the same site for its own degradation. Uhrf1 cannot degraded by UPS and results in accumulation within cells. Specifically, we also evaluate the splicer of Dppa3 and found that the accumulation of Uhrf1 is accompanied with full length of Dppa3 protein level in ES cells. Uhrf1 is better characterized in molecular mechanism in DNA methylation maintenance [35, 45].

## Conclusion

Together, we provide new molecular insight into the function of Dppa3 in ES cells. We identified new functions of Dppa3 in protein stability regulation, Nanog and Uhrf1 regulation in ES cells. Our findings advance our knowledge of the functions and mechanisms of Dppa3 in ES cells, which may contribute to the future application of stem cells in regenerative medicine.

## Supplementary Information

**Additional file 1.** Supplementary Figures and Legends. Supplementary Figures S1-S5. Supplementary Table S1-S3.

## Data Availability

The dataset used and/or analyzed during the current study are available from the corresponding author upon reasonable request.

## Abbreviations

Dppa3: Developmental pluripotency-associated 3
ES cells: Embryonic stem cells
PGCs: Primordial germ cells
IP-MS: Immunoprecipitation-mass spectrometry
ICM: Inner cell mass
Tet3: Ten-eleven translocation 3
iPSCs: Induced pluripotent stem cells
UPS: Ubiquitin–proteasome system
AP: Alkaline phosphatase
CHX: Cycloheximide
