# Supplementary material for: Dppa3 facilitates self-renewal of embryonic stem cells by stabilization of pluripotent factors
Source: Stem Cell Res Ther. 2022 Apr 27;13:169. doi: 10.1186/s13287-022-02846-8 (PMC9044575; doi:10.1186/s13287-022-02846-8)
Supplement: Supplementary file 1 — Additional file 1. Supplementary Figures and Legends. Supplementary Figures S1-S5. Supplementary Table S1-S3. [file 13287_2022_2846_MOESM1_ESM.docx]

**Dppa3 facilitates self-renew of embryonic stem cells by stabilizing of pluripotent factors**

Zhao *et al*.

**Supplemental Figures and Legends**

**Supplemental Figure 1**

**Supplemental Figure 2**

**Supplemental Figure 3**

**Supplemental Figure 4**

**Supplemental Figure 5**

**Supplemental Table 1 *Dppa3* overexpression sequences.**

**Supplemental Table 2 *Dppa3* knockdown sequences.**

**Supplemental Table 3 Primer sequences for qPCR**

**
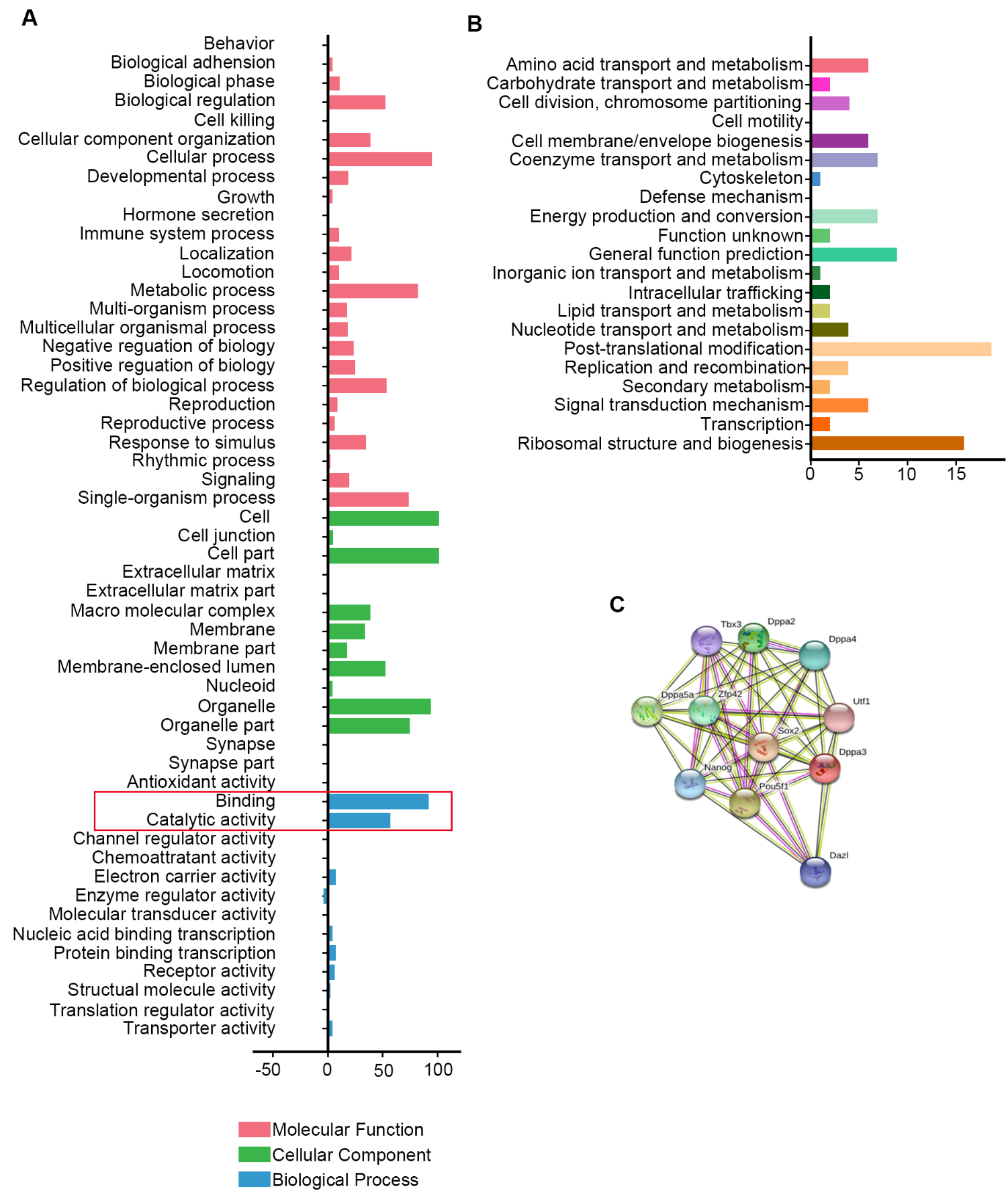
**

**Supplemental Figure 1: The Dppa3-interated proteins are functionally enriched in the ubiquitin proteasome system. A**. GO analysis of Dppa3-interacted proteins **B**. KEGG analysis of Dppa3-interacted proteins. **C**. Prediction of proteins association with Dppa3 using STRING database.

**
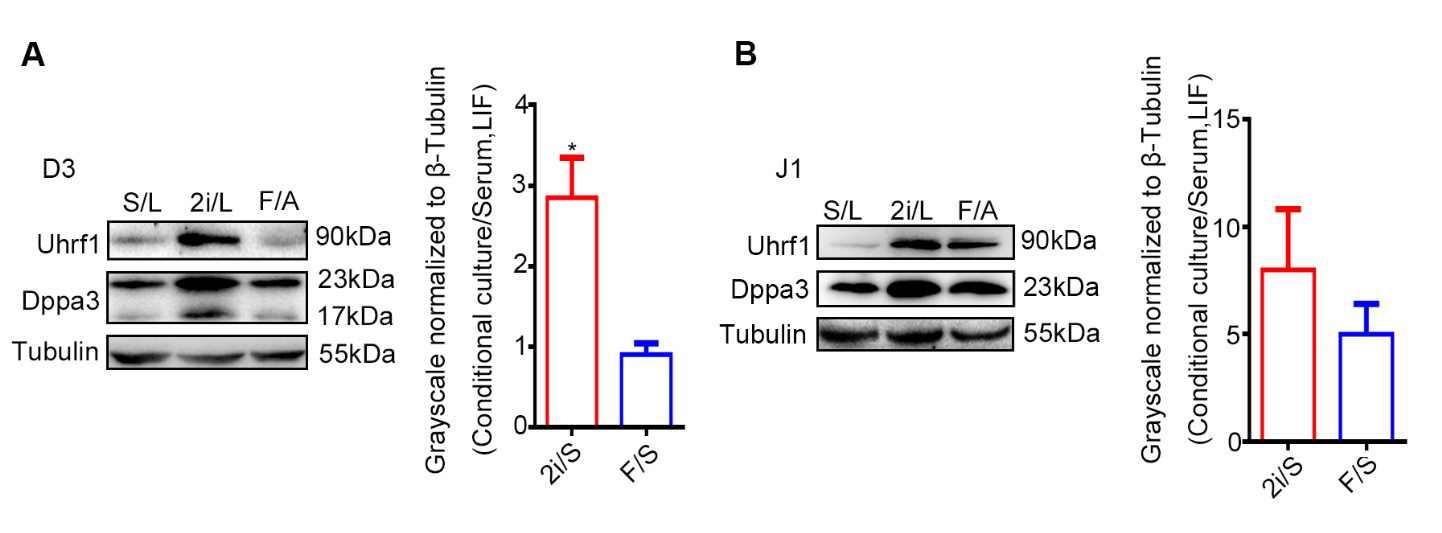
 Supplemental Figure 2: The expression levels of Dppa3 and Uhrf1 were increased in naive state ES cells. A** and **B.** The protein levels of Dppa3 and Uhrf1 were analyzed using western blot in D3 ES cell (**A**) and J1 ES cells (**B**) with different pluripotent state. Naïve pluripotent state was induced using 2i and LIF (2i/L), and the primed state was induced using FGF2 and Activin A (F/A). Quantification of Uhrf1 protein levels normalized to Tubulin by ImageJ software. The data is presented as mean ± SEM. (n=3, *p < 0.05).

**
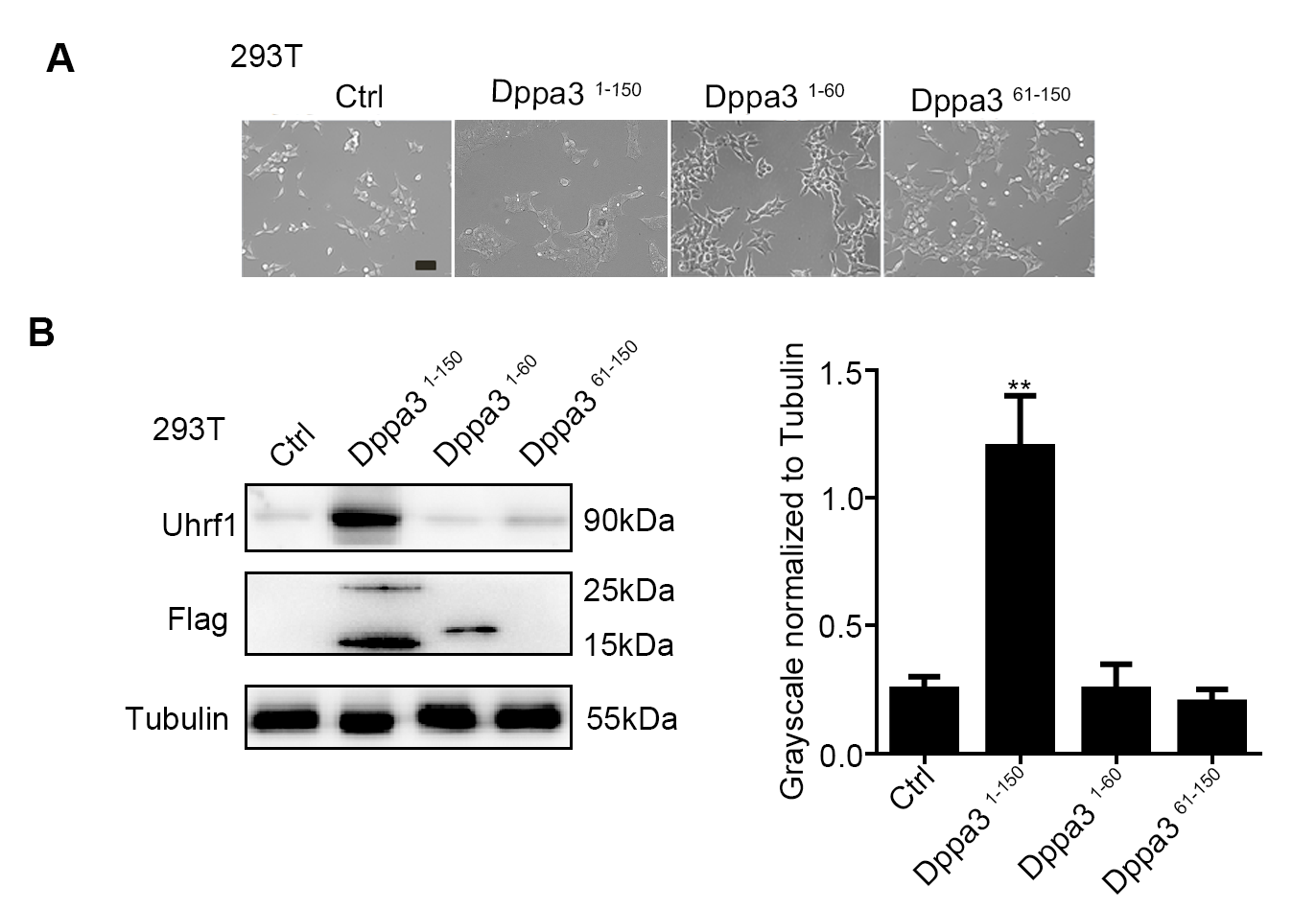
**

**Supplemental Figure 3: Characterization of three different lengths of Dppa3 in 293T cells. A**. Morphology of the 293T cells transfected with three different length of Dppa3. Dppa3 ^1-150^, Dppa3 ^1-60^ and Dppa3 ^61-150^ 293T cells. Scar bar: 100um. **B**. Left panel: The protein levels of Uhrf1 in 293T were analyzed by western blot. Right panel: Quantification of Uhrf1 protein levels by ImageJ software. The data is presented as mean ± SEM. (n=3, ***p* < 0.01).

**
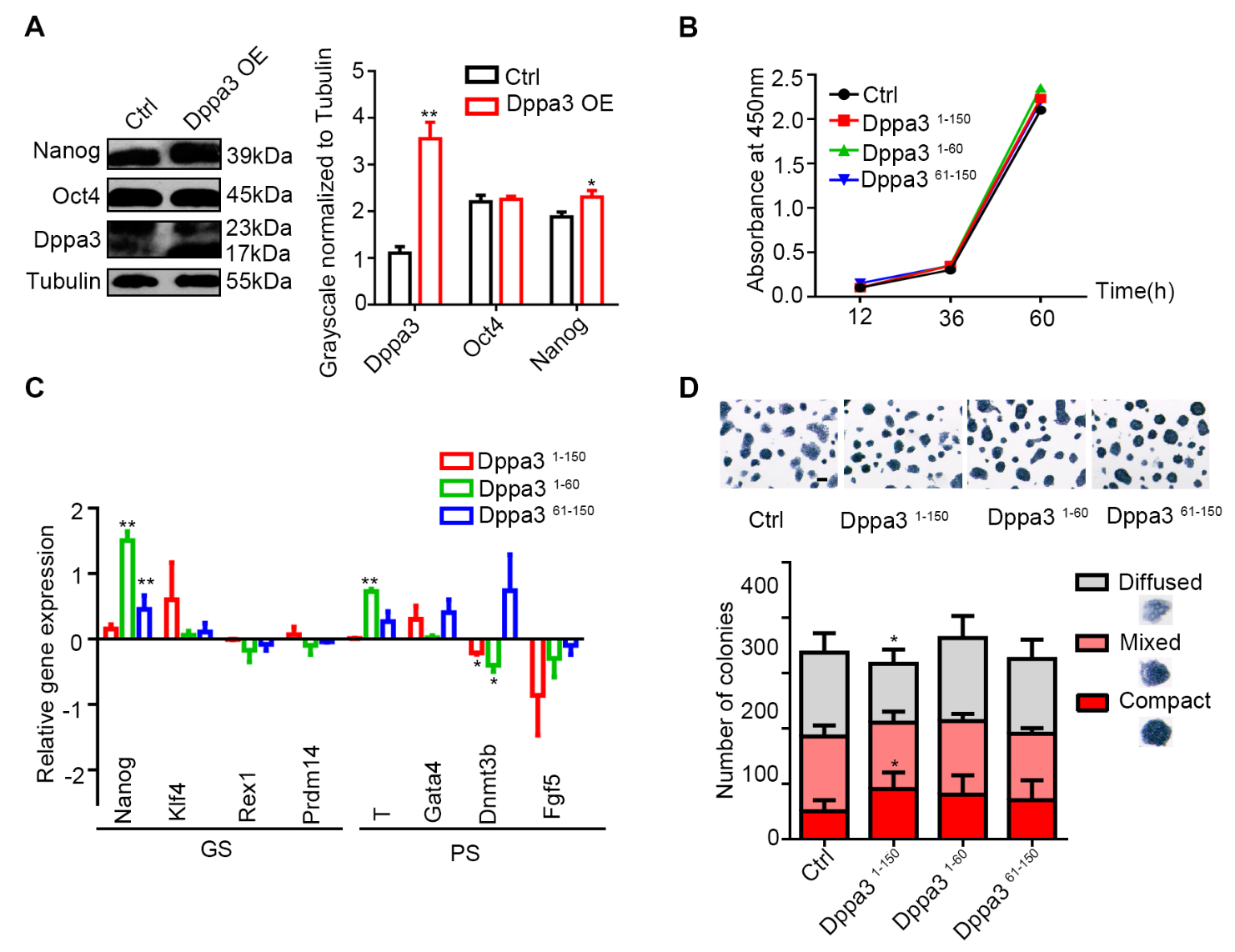
**

**Supplemental Figure 4: Exogenous full length of Dppa3 facilitates the pluripotency maintenance in ES cells.** **A.** Western blot analysis of Nanog and Oct4 levels in Dppa3 overexpression ES cells. Right panel: Quantification of Nanog, Oct4, and Dppa3 protein levels normalized to Tubulin by ImageJ software. The data is presented as mean ± SEM. (n=3, **p* < 0.05, ***p* < 0.01). **B**. Proliferation of control, Dppa3^1-150^, Dppa3^1-60^ and Dppa3^61-150^ ES cells were assessed by CCK-8. Data is presented at least three replications. **C.** Real-time PCR analysis of ground state and primed state-related gene expression in Control, Dppa3^1-150^, Dppa3^1-60^ and Dppa3^61-150^ ES cells. GS, ground state-related genes; PS, primed state-related genes. The data is presented as mean ± SEM (n=3, *p < 0.05, **p < 0.01). **D.** Alkaline phosphatase (AP) staining and quantification of AP-positive colonies of control, Dppa3^1-150^, Dppa3^1-60^ and Dppa3^61-150^ ES cells. Scar bar, 200μm. The data is presented as mean ± SEM. (n=3, *p <0.05).


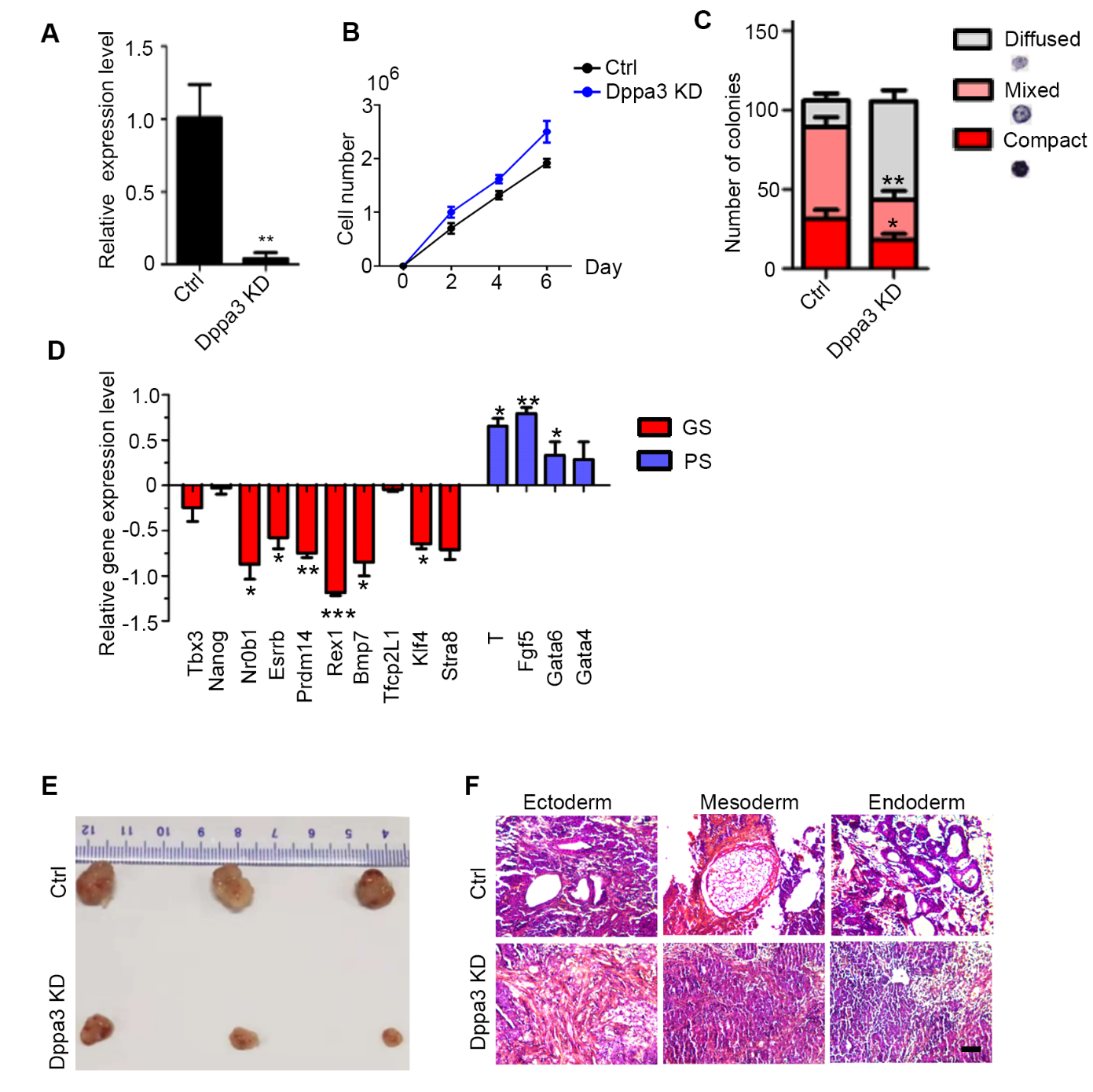


**Supplemental Figure 5: Silencing of Dppa3 in ES cells promotes the pluripotency exit. A.** Relative expression level of Dppa3 in Dppa3 KD and control ES cells. **B.** The proliferation of control and Dppa3 knockdown ES cells were assessed by CCK-8. Data is presented at least three replications. **C.** Quantification of alkaline phosphatase staining (AP) positive colonies of control and Dppa3 KD ES cells. The data is presented as mean ± SEM. (n=3, *p <0.05, ***p* <0.01). **D.** Real-time PCR analysis of ground state and primed state-related gene expression in control and Dppa3 KD ES cells. The data is presented as mean ± SEM (n=3, *p < 0.05, **p < 0.01). **E**. Teratomas derived from Dppa3 knockdown and control ES cells. **F.** HE staining of teratoma formed from Dppa3 knockdown and control ES cells. Scar bar, 100um.

**Supplemental Table 1 *Dppa3* overexpression sequences.**

| **Genes** | **Sequences** |
| --- | --- |
| *Flag*-forward | 5’-TCTCAAGCTTAACTAGCTAGCGGACCGACGC-3’ |
| *Flag* -reverse | 5’-AGAGGTCCCAACCTTATCGTCGTCATCCT-3’ |
| *Dppa3 ^1-150^*-forward | 5’-CCGAGCTAGCTTTTGAGGCT-3’ |
| *Dppa3 ^1-150^*-reverse | 5’-CCCTCGAGATTCTTCCCGATTTTCGCAT-3’ |
| *Dppa3 ^1-60^*-forward | 5’-CCGAGCTAGCTTTTGAGGCT-3’ |
| *Dppa3 ^1-60^*-reverse | 5’-TTTTCTCGAGCCGTAGACTGCGCCGGCGTG-3’ |
| *Dppa3 ^61-150^*-forward | 5’-ATGCTAGCATGAACCGCATTGCAGCCGTA-3’ |
| *Dppa3 ^61-150^*-reverse | 5’-CCCTCGAGATTCTTCCCGATTTTCGCAT-3’ |
| *Dppa3*-forward | 5’-CTTGCTAGCTTTTGAGGCTTCTGCCCATC-3’ |
| *Dppa3*-reverse | 5’-ATTAGATATCCAGCCAGGGCAGCGTACA-3’ |

**Supplemental Table 2 *Dppa3* knockdown sequences.**

| **Genes** | **Sequences** |
| --- | --- |
| siDppa3-F | 5’-GATCCGAAATTGTAGATAGGATGCACATTCAAGAGATGTGCATCCTATCTACAATTTCTTTTTTAAGCTTG-3’ |
| siDppa3-R | 5’-AATTCAAGCTTAAAAAAGAAATTGTAGATAGGATGCACATCTCTTGAATGTGCATCCTATCTACAATTTCG-3’ |

**Supplemental Table 3 Primer sequences for qPCR.**

| **Genes** | **Sequences** |
| --- | --- |
| *Nanog*-forward | 5’-GCTCCGCTCCATAACTTCG-3’ |
| *Nanog*-reverse | 5’-ACCTGGCTTTGCCCTGACT-3’ |
| *Klf4*-forward | 5’-CACCTACCTGGACAAGGTGC-3’ |
| *Klf4*-reverse | 5’-TCCGTCTCAAACTTGGTCCG-3’ |
| *Rex1*-forward | 5’-TCACTGTGCTGCCTCCAAGT-3’ |
| *Rex1*-reverse | 5’-GGGCACTGATCCGCAAAC-3’ |
| *Prdm14*-forward | 5’-TGTGTGGTACGGAAATGGCT-3’ |
| *Prdm14*-reverse | 5’-GGCGTGTACTTCAGGTGCTT-3’ |
| *T*-forward | 5’-CCGGTGCTGAAGGTAAATGT-3’ |
| *T*-reverse | 5’-CCTCCATTGAGCTTGTTGGT-3’ |
| *Gata4*-forward | 5’-GCTATGCATCTCCTGTCACTCAGA-3’ |
| *Gata4*-reverse | 5’-CCAAGTCCGAGCAGGAATTTGAAG-3’ |
| *Gata6*-forward | 5’-CTTCTCCTTCTACACAAGCGACCA-3’ |
| *Gata6*-reverse | 5’-ATACTTGAGGTCACTGTTCTCGGG-3’ |
| *Dnmt3b*-forward | 5’-GCTATTTGTCTTGAGGCGCT-3’ |
| *Dnmt3b*-reverse | 5’-AACTTAGAACCCAGGAGACGC-3’ |
| *Fgf5*-forward | 5’-AGTCAATGGCTCCCACGAAG-3’ |
| *Fgf5*-reverse | 5’-CCACTCTCGGCCTGTCTTTT-3’ |
| *Tbx3*-forward | 5’-GCAGTGGATGTCCAAAGTCGTCACT-3’ |
| *Tbx3*-reverse | 5’-CAGGTAGGTTCGAAAAGTACTGTAA-3’ |
| *Nr0b1*-forward | 5’-ACCGTGCTCTTTAACCCAGA-3’ |
| *Nr0b1*-reverse | 5’-CCGGATGTGCTCAGTAAGG-3’ |
| *Bmp7*-forward | 5’-TACGTCAGCTTCCGAGACCT-3’ |
| *Bmp7*-reverse | 5’-GGTGGCGTTCATGTAGGAGT-3’ |
| *Tfcp2L1*-forward | 5’-AGGTGCTGACCTCCTGAAGA-3’ |
| *Tfcp2L1*-reverse | 5’-GTTTTGCTCCAGCTCCTGAC-3’ |
| *Stra8*-forward | 5’-GTTTCCTGCGTGTTCCACAAG-3’ |
| *Stra8*-reverse | 5’-CACCCGAGGCTCAAGCTTC-3’ |
| *Esrrb*-forward | 5’- CAAGAGAACCATTCAAGGC-3’ |
| *Esrrb*-reverse | 5’- CATCCCCACTTTGAGGCATTT-3’ |
| *18s-*forward | 5’-GTAACCCGTTGAACCCCATT-3’ |
| *18s-*reverse | 5’-CCATCCAATCGGTAGTAGCG-3’ |
